# Diabetic Kidney Disease in Post-Transplant Diabetes Mellitus: Causes, Treatment and Outcomes

**DOI:** 10.3390/biomedicines11020470

**Published:** 2023-02-06

**Authors:** Lee-Moay Lim, Jer-Ming Chang, Hung-Tien Kuo

**Affiliations:** 1 Graduate Institute of Clinical Medicine, College of Medicine, Kaohsiung Medical University, Kaohsiung 807, Taiwan; 2 Division of Nephrology, Department of Internal Medicine, Kaohsiung Medical University Hospital, Kaohsiung Medical University, Kaohsiung 807, Taiwan; 3 School of Medicine, College of Medicine, Kaohsiung Medical University, Kaohsiung 807, Taiwan

**Keywords:** post-transplant diabetes mellitus, kidney transplantation, diabetic kidney disease

## Abstract

Kidney transplant recipients are a unique subgroup of chronic kidney disease patients due to their single functioning kidney, immunosuppressive agent usage, and long-term complications related to transplantation. Post-transplant diabetes mellitus (PTDM) has a significant adverse effect on renal outcomes in particular. As transplantations enable people to live longer, cardiovascular morbidity and mortality become more prevalent, and PTDM is a key risk factor for these complications. Although PTDM results from similar risk factors to those of type 2 diabetes, the conditions differ in their pathophysiology and clinical features. Transplantation itself is a risk factor for diabetes due to chronic exposure to immunosuppressive agents. Considering current evidence, this article describes the risk factors, pathogenesis, diagnostic criteria, prevention strategies, and management of PTDM. The therapeutic options are discussed regarding their safety and potential drug–drug interactions with immunosuppressive agents.

## 1. Introduction

The transplantation of kidneys is a treatment option for end-stage kidney disease (ESKD). As a result of the transplantation, the patient received a new kidney, but this also triggered the process of chronic kidney disease (CKD). As a subset of CKD patients, kidney transplant recipients (KTR) continue to have a high mortality rate and risk of dialysis progression. CKD progression in KTR involves a complex network of allogeneic and non-allogeneic risk factors, including hypertension, diabetes, proteinuria, dyslipidaemia, and bone mineral disorder [1].

Comorbidities after kidney transplantation may affect short-term and long-term transplant outcomes. In recent years, short-term grafts and patient survival of kidney transplants have improved dramatically due to improvements in surgical techniques and the development of new immunosuppressive agents [2,3]. Kidney transplant recipients have a higher risk of cardiovascular disease, infection, and malignancies compared with the general population [4]. Special consideration should be given to post-transplant diabetes mellitus (PTDM). PTDM is commonly associated with metabolic disorders or infections and its potential to cause diabetic kidney disease, nephrotic syndrome, and the loss of allografts as well as to impair renal function [5,6].

The term PTDM was adopted in 2014 after a consensus meeting at which it was decided that the time of diagnosis rather than the time of occurrence would be adopted [7]. The purpose of the change in terminology was to exclude transient post-transplant hyperglycemia from PTDM diagnoses. Since 2003, PTDM is known as new-onset diabetes after transplantation (NODAT) [5]. According to the consensus reached in 2014, an accurate diagnosis of PTDM can be made once a patient’s clinical condition is stable following the adoption of maintenance immunosuppressive treatment, the stabilization of allograft kidney function, and the elimination of acute infection [7]. A study using data from the United States Renal Data System(USRDS) showed that PTDM was associated with an increased risk of transplant failure (relative risk (RR): 1.63, 95% confidence interval [CI]: 1.46–1.84, *p* < 0.0001), death-censored transplant failure (RR: 146, 95% CI: 1.25–1.70, *p* < 0.0001), and mortality (RR: 1.87, 95% CI: 1.60–2.18, *p* < 0.0001) during a median 3-year follow-up period [8]. Prevention, early diagnosis, and appropriate treatment of PTDM are important issues in the care of kidney transplantation recipients.

## 2. Review Protocol

This review was designed according to the Preferred Reporting Items for Systematic Reviews and Meta-Analyses (PRISMA) guidelines [9]. In brief, we searched Pubmed and Google scholar up to 1 November 2022 using the following keywords: diabetes mellitus (DM), type 2 diabetes mellitus, post-transplant diabetes mellitus, kidney transplantation, transplant outcomes, immunosuppression, calcineurin inhibitors, mTOR inhibitor, steroid, risk factors, treatment, dipeptidyl peptidase 4 inhibitors, glucagon-like peptide-1 receptor agonists, and sodium–glucose cotransporter 2 inhibitors. We applied inclusion and exclusion criteria as follows: full text, meta-analysis, randomized control trial (RCT), review, systematic reviews, humans only, English. The collected literature was agreed upon by all authors and was included for further review.

## 3. Risk Factors for PTDM

PTDM is a clinical entity distinct from type 1 and type 2 DM. The pathogenesis of PTDM is multifactorial, which is based on β-cell dysfunction in the presence of insulin resistance. Diabetogenic immunosuppressive medication may determine the occurrence of PTDM in a high-risk patient [10]. Major risk factors for PTDM are summarized in Table 1. Traditional risk factors for diabetes mellitus (DM) in the general population, such as family history, age, obesity, and ethnicity, are also associated with the development of PTDM in kidney transplant recipients [8]. Among these, obesity is a potentially modifiable factor. Among patients with CKD, central obesity is a modifiable risk factor for the progression of kidney disease [11]. Compared with normal-weight people (body mass index [BMI] 18.5–25), overweight people (BMI > 30) have an elevated risk of CKD (RR: 1.40; 95% CI: 1.30–1.50), and obese people (BMI > 30) are at an even higher risk (RR: 1.83, 95% CI: 1.57–2.13) [12]. The prevalence of obesity among kidney transplant recipients has increased in recent decades. In the United States, the prevalence of obesity among kidney transplant recipients increased from 20% in the 1990s to 30% in 2011 [13]. Obesity may lead to adipose tissue dysfunction, insulin resistance, and impaired glucose metabolism [14]. Obesity has been associated with PTDM in both adult and child recipients of kidney transplants [8,15].

Viral infections may also increase the risk of PTDM. An association of hepatitis C virus (HCV) infection with DM has been shown in the general population and among organ transplant recipients [8,16]. The association between type 2 diabetes and chronic hepatitis C has been shown in several epidemiological studies. HCV infection leads to DM through direct viral effects, insulin resistance, proinflammatory cytokines, chemokines, and other immune-mediated mechanisms [17]. In the past, HCV treatment with interferon and ribavirin was associated with a high recurrent rate [18], and the risk of acute rejection after interferon treatment was a major concern in kidney transplant recipients. At present, HCV can be eradicated effectively with direct antiviral agents (DAAs) [19] before or after kidney transplantation. Hence, the effect of HCV infection on PTDM risk may be modified by treatment with DAAs.

The incidence of PTDM was higher in patients with cytomegalovirus (CMV) infection than in those without CMV infection [20]. In the era before prophylaxis, the incidence of CMV infection was high. CMV infection contributes to mortality and morbidities including PTDM in kidney transplant recipients. CMV infection may lead to the destruction of pancreatic β-cells or the production of pro-inflammatory cytokines, but further studies are required to investigate these mechanisms [21]. With the introduction of policies promoting the prophylactic treatment of CMV in high-risk patients, the incidence of CMV infection after kidney transplantation has been effectively reduced [22]. The effect of CMV infection on PTDM risk may also be modified by CMV prophylaxis [20].

**Figure 1:**
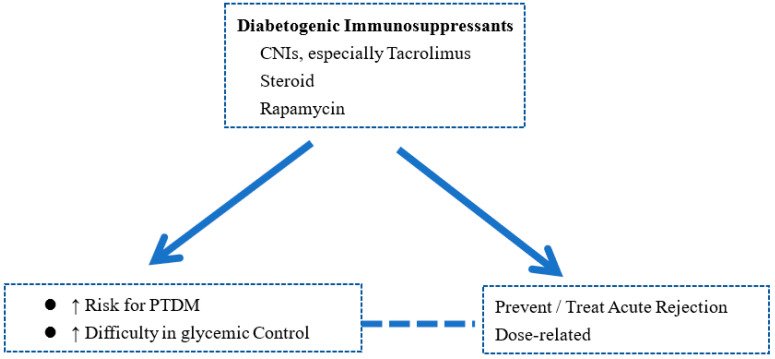
Inter-relations of Rejection, Immunosuppression, and Diabetes.

The development of PTDM is strongly related to immunosuppression and acute rejection (Figure 1). Acute rejection significantly affects the outcomes of long-term transplant. Acute rejection, human leukocyte antigens (HLA) mismatch, and retransplantation are well-known predictors of PTDM. Furthermore, immunosuppressive agents such as steroids, calcineurin inhibitors (CNI), and even mammalian target of rapamycin (mTOR) inhibitors are potentially diabetogenic and may lead to PTDM. The association between steroids and hyperglycemia is well known. The mechanisms include increased gluconeogenesis, increased insulin resistance, reduced glycogenesis, decreased insulin release, and impaired pancreatic beta cell function [23]. CNI, especially tacrolimus, are associated with an increased risk of PTDM [24]. Tacrolimus may lead to PTDM by reducing glucose uptake, decreasing insulin release, reducing insulin gene expression, and direct pancreatic β-cell toxicity [25]. The association of mTOR inhibitors with PTDM has also been indicated [26,27]. The diabetogenic effects of mTOR inhibitors may be mediated by hyperglycemia and the inhibition of pancreatic β-cell proliferation [25].

The impact of major immunosuppressants on PTDM development has been compared by prospective trials, meta-analysis, and analysis of registered database. Evidence shows that tacrolimus is associated with an increased risk of PTDM compared to cyclosporine. In a randomized prospective trial by Vincenti F et al., PTDM or impaired fasting glucose occurred in 73 of 336 cyclosporine patients (26.0%) and 96 of 346 tacrolimus patients (33.6%, *p* = 0.046) at 6 months after kidney transplantation [28]. In an analysis using data from the USRDS, the use of tacrolimus as the initial maintenance immunosuppressive medication was associated with a higher risk of PTDM (relative risk 1.53, *p* < 0.0001) [8]. In a retrospective analysis by Araki M et al., the incidence of PTDM needing insulin for cyclosporine, tacrolimus, and sirolimus was 7.6%, 11.7%, and 5.9%, respectively, in a mean follow up of 39.2 months. In multivariate analysis, tacrolimus usage (hazard ratio 3.794, *p* = 0.002) and pulse steroid therapy for acute rejection (hazard ratio 2.491, *p* = 0.0115) were independent predictors for PTDM development [29]. In a recent meta-analysis by Kotha et al., they estimated the relative effects of tacrolimus, cyclosporine, and sirolimus on PTDM development and concluded that sirolimus and tacrolimus were significantly more diabetogenic than cyclosporine. Tacrolimus has higher diabetogenicity in the short term (2–3 years post-transplant), whereas sirolimus tends to exhibit higher diabetogenicity in the long-term (5–10 years post-transplant) [30].

PTDM may be a consequence of intensive immunosuppression used to prevent or treat acute rejection. Conversely, acute rejection may occur as a result of decreases in the dosage of immunosuppressive agents in order to avoid or treat PTDM. The balance between immunosuppression minimization, acute rejection, and PTDM needs to be carefully considered individually.

## 4. Pathophysiology of Diabetic Kidney Disease in PTDM

Diabetic kidney disease (DKD), also known as diabetic nephropathy (DN), is defined as an increase in urine albumin excretion or a decrease in the glomerular filtration rate (GFR) or both [31]. Worldwide, approximately 40% of patients with diabetes develop DKD and subsequently progress to kidney replacement therapy such as dialysis and kidney transplantation [32,33]. A complex network of pathological changes occured as a result of hemodynamic and metabolic abnormalities. The expansion of the mesangium, thickening of the glomerular basement membrane (GBM), glomerulosclerosis, and presence of podocyte injury are the morphological changes that are typically found with kidney biopsies [34,35].

A change that is observable by electronic microscopy at the early stage of diabetes is thickening of the GBM, which can be detected as early as 2 years after the onset of type I DM [36]. A linear relationship exists between diabetes duration and the width of the GBM in patients with type I DM [37]. By contrast, mesangial expansion begins in the 5th year after the onset of type I DM [38]. The onset of these morphological changes in glomerular architectures is different in type II diabetes mellitus; the time of onset is usually unknown in patients with type II DM. Among patients with DM (type I or II), the mesangial fractional volume is associated with the albumin excretion and glomerular filtration rates [39,40]. As the disease progresses and mesangial expansion continues, Kimmelstiel–Wilson nodules are formed, resulting in nodular accumulations of the mesangial matrix [41]. In contrast to patients with diffuse mesangial expansion, those with nodular diabetic glomerulosclerosis present with more severe renal damage, a longer diabetic duration, and a poorer renal outcome [42].

A number of extraglomerular lesions are closely related to renal function loss during the progression of ESKD, including tubular atrophy, interstitial inflammation, and tubulointerstitial fibrosis [43]. As part of DN, hyalinosis often occurs in efferent arterioles [44]. DN affects all kidney structural components and has a wide range of pathological manifestations. A renal biopsy could allow the diagnosis of these lesions and their morphological characteristics, thus preventing, slowing down, or even reversing DN.

According to reports, PTDM is most likely to develop in the first year after kidney transplantation and has been associated with poor graft survival and poor graft quality [8,45,46]. Approximately 7–30% of patients must start blood glucose management in addition to their immunosuppressive agents [6,47]. Several studies have discovered associations between PTDM and premature cardiovascular disease and mortality in kidney transplant recipients [48,49]. PTDM has many of the same characteristics as type II DM, including insulin resistance, hypertriglyceridemia, hypertension, low-grade inflammation, obesity, and decompensated insulin release [50,51]; however, the underlying mechanisms might be different. Defects in glucose homeostasis mediated by the fine tuning of insulin secretion is the primary mechanism leading to type II DM [50]. Diminished insulin-mediated glucose uptake in peripheral tissue [52,53], impairment in the insulin-mediated suppression of hepatic glucose output [53], dysfunction in insulin release [54], and impairment of the incretin axis between the gut and pancreas [55], which reinforces impaired B-cell function and increases glucagon production, are the common pathogenic mechanisms in both patients with type II DM and those with PTDM. However, renal gluconeogenesis and increased proximal tubular sodium–glucose reabsorption have not been demonstrated in patients with PTDM [47].

## 5. Diagnosis of DKD and PTDM

PTDM had been diagnosed on the basis of the level of insulin required, type of oral hypoglycemic agents administered, and level of fasting plasma glucose posttransplant; these diagnostic criteria were used to distinguish between PTDM and pre-existing DM, and PTDM was significantly underdiagnosed [47]. The distinction between PTDM and other forms of posttransplant hyperglycemia, such as those caused by medication or anxiety, is important for clinical reasons. In 2003, international guidelines on the diagnostic criteria of PTDM were updated to include a 2 h oral glucose tolerance test (OGTT) in accordance with World Health Organization (WHO) guidelines [56]. The definition and diagnosis of PTDM should be based on the following criteria: (1) random glucose ≥ 200 mg/dL (11.1 mmol/L) with symptoms, (2) fasting glucose ≥ 126 mg/dL (7 mmol/L), and (3) a 2 h OGTT ≥ 200 mg/dL (11.1 mmol/L) [56]. A decision was made by the American Diabetes Association and the WHO in 2011 to include HbA1c measurement as part of the diagnosis of PTDM [57]. Even so, this criterion might not be uniformly applied to the diagnosis of PTDM due to the heterogeneity in HbA1c levels in the early post-transplant period, at which time HbA1c levels may be compromised by anemia and bone marrow production may be suppressed due to immunosuppressive agents [58]. At the international consensus meeting on PTDM in 2014, it was agreed that HbA1c levels should not be used as the sole indicator to diagnose the disease [7]. For diagnosing PTDM, the OGTT is currently the gold standard [7]. OGTTs must not be performed sooner than 2 months after transplantation or before a patient’s clinical condition has stabilized following the adoption of immunosuppressive agents [47].

## 6. Prevention of PTDM

Preventing PTDM starts with identifying the risk factors before transplantation. Kidney transplant candidates must undergo a thorough evaluation of their diabetes risk prior to enlistment. Preventive strategies such as weight reduction, lifestyle modification, caloric control, and physical exercise should be recommended to high-risk patients. For patients at greater risk of PTDM, such as those aged >45 years [59] or those with a family history of diabetes, metabolic syndrome with hypertriglycemia [60], hypertension, impaired fasting glucose [61], or HCV infection [62,63], HbA1c levels should be measured and OGTTs should be performed [5,47]. However, HbA1c levels are not a good indicator of blood glucose during the early post-transplant period due to bleeding, bone marrow suppression, or the use of erythropoietin in the case of delayed graft function [58]. Jenssen T et al. suggested that fasting plasma glucose should be kept <7 mmol/L (126 mg/dL) and postprandial glucose should be <10 mmol/L (180 mg/dL) in the early post-transplant period [50]. HbA1c levels of 7.0–7.5% (53–58 mmol/mol) are generally recommended [7,64]. Bergrem H et al. performed OGTTs in a retrospective cohort study involving 301 patients without pretransplant DM and discovered that the 2 h plasma glucose level was the strongest predictor of PTDM [61].

Immunosuppressive agents such as glucocorticoids and CNI are well known to cause PTDM [47]. Immunosuppressive agents without diabetogenic effects are recommended for patients at high risk of PTDM [65,66]. Various strategies are available for modifying immunosuppression, including reducing the dose or discontinuing glucocorticoids, adding azathioprine or mycophenolate to decrease the CNI dosage, and replacing CNI with less diabetogenic medication, such as cyclosporine or an mTOR inhibitor [5]. A randomized controlled trial conducted by Torres A. et al. revealed that cyclosporine may reduce the risk of PTDM compared with tacrolimus despite the fact that cyclosporine is a diabetogenic drug [67]. Belatacept, a costimulation blocker acting on CD28–CD80/86 pathways, can be used as an alternative treatment for patients who have developed PTDM receiving CNI [68,69]. Santos A H et al. discovered that a tailored immunosuppression regimen based on HCV or CMV serology may reduce the risk of PTDM [70]. While tapering or tailoring the immunosuppressive dose, the risk of rejection and graft failure must be carefully considered.

Vitamin D is well known for its role in regulating bone health and calcium metabolism. Recent studies have shown that vitamin D is involved in a wide range of pleiotropic functions mediated through vitamin D receptors [71], such as the modulation of cell growth and differentiation [72] and the regulation of immune functions [73], providing a protective effect against diabetes and cardiovascular disease [74]. In kidney transplant recipients, vitamin D may regulate the immune response and protect against cardiovascular disease, cancer, and infection [75]. Patients receiving kidney transplantation are at increased risk for hypovitaminosis D due to the fact that they are typically advised to avoid sun exposure in order to prevent the development of skin malignancy related to their immunosuppressive treatment [76]. Hypovitaminosis D has been associated with PTDM risk in several studies [77,78]. Le Fur A et al. showed that 25(OH)D deficiency (≤10 ng/mL) at the time of transplant was an independent risk factor for PTDM in the first year after kidney transplant (hazard ratio: 2.41, 95% CI: 1.01–5.75, *p* = 0.048) [79]. In a multi-center cohort study of 442 kidney transplant recipients, Quach et al. discovered that lower serum 25(OH)D was associated with a higher risk of PTDM [77]. At present, there is limited evidence linking the role of vitamin D deficiency and PTDM from in vivo or in vitro studies. We believe that the role of vitamin D is an encouraging point to be taken into consideration in battling against PTDM. Vitamin D supplementation may offer protection against PTDM in high-risk patients.

## 7. Management of DKD in Patients with PTDM

### 7.1. Lifestyle Modification

Lifestyle modifications that promote fat and energy expenditure, moderate-intensity physical activity, and moderate weight loss are strategies that can be followed to reduce the risk of type 2 diabetes mellitus [80,81]. In a randomized controlled trial (DIADEM-I) performed in primary care and community settings, Taheri S et al. discovered that intensive lifestyle interventions resulted in significant weight loss at 12 months, diabetes remission in more than 60% of participants, and normoglycemia in more than 30% of participants [82]. Nonetheless, evidence of the effect of lifestyle interventions on the risk of PTDM is lacking. In a randomized controlled trial regarding glycemic control strategies involving 103 kidney transplant recipients, active lifestyle intervention led by renal dietitians did not improve surrogate markers of glucose metabolism [83]. In addition, small-scale studies have recently reported favorable outcomes from moderate-to-vigorous physical activity, a Mediterranean-style diet, and a plant-based diet on the risk of PTDM [84,85,86]. In a randomized controlled trial (CAVIAR) comparing active versus passive lifestyle intervention post-kidney transplantation, there was no influence of active versus passive lifestyle intervention on surrogate glucose metabolism measurement although there were improvements in secondary clinical endpoints including weight loss, reduced fat mass, and a suggestion of reduced PTDM incidence (7.6% versus 15.6%, respectively, *p* = 0.123) [83]. Further studies are needed to determine the benefits of lifestyle modification after kidney transplantation. Management of PTDM is a challenging task due to the fluctuation in kidney function in different stages after organ transplantation. Clinicians must consider the potential adverse effects of pharmacological antidiabetic treatment before introducing this therapy to patients.

### 7.2. Medication Treatment

#### 7.2.1. Insulin

In the first few months following transplantation, stress hyperglycemia and immunosuppressive drugs frequently cause high blood sugar levels [87,88]. One of the major pathophysiological features is impaired insulin secretion. Preservation of β-cell function should be undertaken as soon as possible after a diagnosis of diabetes is given to prevent β-cell glucotoxicity and overstimulation [47,89]. At this early stage, insulin is the most effective and safest prescription. Hecking M et al. revealed in their pilot study that basal insulin supplementation especially during the first week after kidney transplantation resulted in significantly fewer cases of PTDM as a result of the insulin-mediated protection of β-cells [89]. However, in a recent multicenter randomized controlled trial involving the aggressive intervention of blood sugar control using insulin in the early postoperative phase of kidney transplantation, no conclusive results were observed [90]. The use of antihyperglycemic agents instead of insulin is the preferred treatment method for patients with mild hyperglycemia after transplantation.

#### 7.2.2. Metformin

Recently, the Association of British Clinical Diabetologists and the British Renal Association released guidelines for the detection and management of diabetes following solid organ transplantation. The guidelines suggested the stratification of pharmacological therapies and recommended metformin as the first-line oral therapy if eGFR ≥ 30 mL/min/1.73 m^2^ and BMI ≥ 25 kg/m^2^ in kidney transplant recipients [91]. In patients with PTDM who are overweight and have stable renal function and no other contraindications, metformin therapy should be recommended [91]. Although metformin reduces glucose levels, it increases insulin sensitivity, promotes weight reduction, may have antineoplastic effects, and endorses cardiovascular health. The use of metformin to treat PTDM is limited because its safety in patients following organ transplant has not been demonstrated. In a retrospective cohort study conducted by Stephen J et al. that examined data from the Scientific Registry of Transplant Recipients, pharmacy claims for a metformin-containing product were not associated with worse patient or allograft survival in kidney transplant recipients [92]. In a subsequent study by Vest LS et al., metformin-based diabetes treatment regimens were not associated with worse graft or patient outcomes [93]. Thus, the use of metformin may be safe in carefully selected kidney transplant recipients [93]. In a pilot randomized controlled trial assessing the feasibility, tolerability, and efficacy of metformin after renal transplantation in patients with impaired glucose tolerance, no serious adverse events were reported in the treatment cohort [94]. However, no difference in metabolic outcomes in their patients was observed [94]. In summary, the efficacy and safety of metformin after renal transplantation are yet to be confirmed; large randomized controlled trials are warranted.

#### 7.2.3. Dipeptidyl Peptidase 4 Inhibitors

Dipeptidyl peptidase 4 inhibitors (DPP4i) are selective inhibitors of DPP4 that have been linked to the degradation of two major incretins, glucagon-like peptide 1 and glucose inhibitory peptide 2 [95]. Weight gain and hypoglycemia have not been associated with DPP4i, making them useful as an antidiabetic agent [95]. Studies have demonstrated potential pleiotropic effects of DPP4i on the heart, blood vessels, and kidney that are independent of hypoglycemic effects, including anti-inflammatory, antihypertensive, and antiapoptotic effects [96]. Furthermore, DPP4i have the ability to promote beta cell proliferation, neogenesis, and the inhibition of apoptosis [97]. This is particularly beneficial in cases of PTDM; DPP4i may protect against CNI toxicity toward beta cell mass and increase islet apoptosis [98]. In a study involving patients with PTDM, Strom Halden TA et al. found that insulin sensitivity was higher during the administration of sitagliptin (25.3% (1.6–49.9%, *p* = 0.04) [99].

Early DPP4i administration may reduce insulin resistance in patients with PTDM. In a single-center study, Thiruvengadam S et al. discovered that the introduction of linagliptin at a median of 90 days post-transplant improved the calculated homeostatic model assessment of insulin resistance (HOMA-IR) score compared with the conventional treatment with insulin [100]. Because DPP4i are well tolerated with only minimal gastrointestinal side effects, they are being utilized more frequently to treat PTDM [101,102]. Except for sitaglipin and valdagliptin, DPP4i do not adversely affect CNI levels [103]. In addition to being PGP substrates, both sitaglipin and valdagliptin are at a higher risk of interacting with cyclosporine [103,104]. The combined administration of cyclosporine and sitagliptin resulted in a 28% increase in sitagliptin AUC [105].

#### 7.2.4. Glucagon-like Peptide-1 Receptor Agonists

Glucagon-like peptide-1 receptor agonists (GLP-1 RA) are beneficial in the treatment of PTDM in terms of improving insulin secretion and normalizing glucagon production [55]. GLP-1 RA cause fewer hypoglycemic episodes [106], a higher reduction of HbA1c [107], and a greater weight loss effect [108,109]. As discussed in a previous section, obesity is a strong predictor for the development of PTDM, and obesity is associated with an increased risk for cardiovascular disease in diabetic patients. In the general population, type 2 DM is closely linked to “android obesity”, characterized by abdominal visceral fat accumulation [110,111]. In a study by Rondanelli M et al., 24 weeks of GLP-1 RA liraglutide treatment led to the reduction of fat mass, android fat, trunk fat, and appetite by improving the lipid profile, glucose control, and insulin sensitivity [112]. In addition to delaying gastric emptying, GLP-1 RA may cause increased gastrointestinal side effects, including nausea, vomiting, and diarrhea, affecting the stability of CNI drugs [107]. A possible association between pancreatitis and malignancy has been noted, but no convincing evidence has been forthcoming [5]. In a randomized controlled trial, the protective potential of GLP-1 RA on the heart and kidney in patients who had not undergone organ transplantation was demonstrated [113]. Few randomized controlled trials have evaluated GLP-1 RA for use in treating PTDM; most studies were case series or retrospective analyses. The largest study in the literature thus far by Singh P et al. compared the efficacy of two GLP-1 RA, dulaglutide and liraglutide, in the management of diabetes after solid organ transplantation [114]. A sustained reduction in weight, BMI, insulin requirement, and HbA1c with mild improvement in serum creatinine and estimated GFR (eGFR) in the arm with dulaglutide was reported [114]. The generalizability of these findings may be limited by their retrospective and observational study designs.

#### 7.2.5. Sodium–Glucose Cotransporter 2 Inhibitors

Sodium–glucose cotransporter 2 (SGLT2) inhibitors inhibit glucose resorption in the renal proximal tubule and lead to glycosuria [115]. In addition, SGLT2 inhibitors block proximal sodium reabsorption, lead to natriuresis, and increase the delivery of sodium to the macula densa [116]. Increased sodium delivery to the macula densa normalizes tubuloglomerular feedback and thereby reduces intraglomerular pressure through constriction of the abnormally dilated afferent arteriole [116]. In addition to their sugar lowering effect, SGLT2 inhibitors reduce intravascular volume and blood pressure [117]. The outcomes of randomized prospective trials have shown that SGLT2 inhibition reduces the likelihood of major adverse cardiovascular events and also reduces mortality, the rate of hospitalization for heart failure, and the progression of DKD [118,119]. The effects of SGLT2 inhibitors in renal transplant recipients are still unclear. In published reports, SGLT2 inhibitors have been effective in glucose and body weight control among transplant recipients with good graft function [120,121]. In a meta-analysis by Chewcharat et al. that included eight studies with 132 patients (baseline eGFR 64.5 ± 19.9 mL/min/1.73 m^2^) treated with SGLT2 inhibitors, SGLT2 inhibitors significantly lowered HbA1c (−0.56% [95% CI: −0.97 to −0.16], *p* = 0.007) and body weight (−2.16 kg [95% CI: −3.08 to −1.24], *p* < 0.001) relative to baseline levels [122]. However, no significant changes in eGFR, the urine protein creatinine ratio, or blood pressure were observed [122]. The effects of SGLT2 inhibitors in recipients with impaired graft function remain unclear. The long-term renal and cardiovascular protective effects of SGLT2 inhibitors in renal transplant recipients require further investigation with randomized prospective trials.

There are potential complications of SGLT2 inhibitor in KTR. The glycosuric effect of SGLT2 inhibitors contributes to the side effect for urinary tract infection in non-transplant population. The altered genitourinary anatomy and immunosuppressive status in KTR may further increase the risk of severe urinary tract infection. In addition, SGLT2 inhibitors may carry risks for hypotension and acute kidney injury. In the immediate and early post-transplant period, KTR are predisposed to ischemic-reperfusion injury and the high intensity of the immunosuppressant used. The risk and benefit of SGLT2 inhibitor usage in the early post-transplant period should be carefully evaluated. Data in non-transplant patients also suggested that SGLT2 inhibitors may carry risks for euglycemic ketoacidosis [123]. To avoid this complication, the use of SGLT2 inhibitors in KTR should be avoided in cases of insulin deficiency and during episodes of acute illness [123].

## 8. Conclusions

Kidney transplant recipients are a subset of patients with CKD that remain at higher risk of CKD complications. In addition to long-term complications, PTDM can result in graft loss and mortality. The early identification of modifiable risk factors and timely implementation of prevention strategies such as lifestyle modifications and immunosuppressive agent adjustments are crucial parts of an overall post-transplant care program. While strategies involving the lowering of immunosuppressive agents reduce the PTDM risk, rejection rates may be higher as a result. To achieve optimal long-term outcomes, a care plan with a patient-specific regimen design and multidisciplinary care involving diabetes specialists and members of the transplant teams should be applied following kidney transplant.

## Figures and Tables

**Table 1:** Major risk factors of PTDM and strategy of prevention.

	Modifiable	Strategy of PTDM Prevention
Traditional risk factors for DM		
Family history	No	
Old age	No	
Obesity	Yes	Body weight control
Black race	No	
Virus		
Hepatitis C	Yes	DAA treatment before transplant
CMV	Yes	CMV prophylaxis
Transplant-associated factors		
Retransplant	No	
High level of HLA mismatch	No	
Acute rejection	Yes	1. Induction therapy
		2. Balance between diabetogenic immunosuppressants and rejection prevention.
Immunosuppressive agent	Yes	
No induction		Induction in risky patient
CNI, especially tacrolimus		CNI minimization/elimination
Steroid		Steroid withdrawal
mTORi?		

Abbreviation: HCV, hepatitis C; DAA, direct antiviral agent; CMV, cytomegalovirus; HLA, human leukocyte antigens; CNI, carcineurin inhibitor; mTORi, mammalian target of rapamycin inhibitors.

## Data Availability

Not applicable.
